# A Chinese Character Teaching System Using Structure Theory and Morphing Technology

**DOI:** 10.1371/journal.pone.0100987

**Published:** 2014-06-30

**Authors:** Linjia Sun, Min Liu, Jiajia Hu, Xiaohui Liang

**Affiliations:** 1 State Key Laboratory of Virtual Reality Technology and Systems, School of Computer Science and Engineering, Beihang University, Beijing, China; 2 School of Chinese Language and Literature at Beijing Normal University, Beijing, China; Xiamen University, China

## Abstract

This paper proposes a Chinese character teaching system by using the Chinese character structure theory and the 2D contour morphing technology. This system, including the offline phase and the online phase, automatically generates animation for the same Chinese character from different writing stages to intuitively show the evolution of shape and topology in the process of Chinese characters teaching. The offline phase builds the component models database for the same script and the components correspondence database for different scripts. Given two or several different scripts of the same Chinese character, the online phase firstly divides the Chinese characters into components by using the process of Chinese character parsing, and then generates the evolution animation by using the process of Chinese character morphing. Finally, two writing stages of Chinese characters, i.e., seal script and clerical script, are used in experiment to show the ability of the system. The result of the user experience study shows that the system can successfully guide students to improve the learning of Chinese characters. And the users agree that the system is interesting and can motivate them to learn.

## Introduction

**Figure 1:**
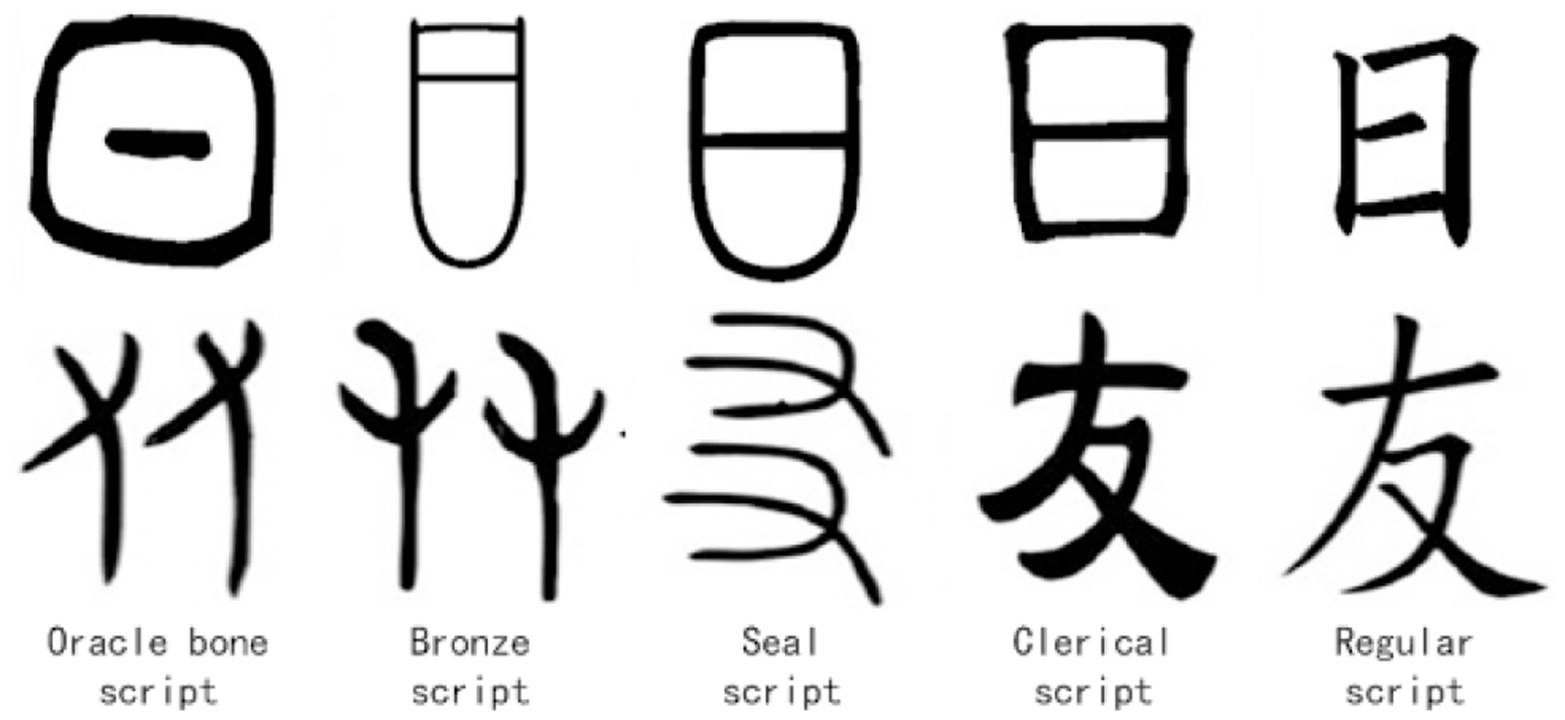
The different scripts of Chinese character.

Chinese character is well known as an ideographic and hieroglyphic system, which is the oldest writing system and is still used at present. For example, the Chinese character “
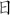
” denotes that an active energy source represented by a dot manifest in the space. During the development history, Chinese characters experienced different writing stages and there are five major scripts, i.e., oracle bone script, bronze script, seal script, clerical script and regular script. As shown in Figure 1, the evolution of “
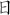
” in the five major scripts could have been influenced by the introduction of the brush as a writing tool, which made lines easier to draw than circles. These scripts are intrinsically related, although the shape and the topology have had the huge evolutions. If understanding the process of evaluation, it would be very help for systematically learning the Chinese character [1].

In this paper, a Chinese character teaching system is proposed to show the evolutions, which are constructed based on the Chinese character structure theory and the 2D contour morphing technology. The Chinese character structure theory provides the systematically theoretical foundation for describing Chinese characters [1]. In this theory, the elements of Chinese character writing system are not characters but components which bring certain meanings, when they were used to compose characters. After many years of research, the experts have obtained a lot of data of Chinese characters at different writing stages, and systematically sorted out the component database of Chinese character at different writing stages. For example, there are 543 components in seal script, 527 components in clerical script and 514 components in regular script, respectively [1]–[3]. Based on the Chinese character structure theory, the system firstly divides every Chinese character into the set of components. Then, the correspondence relationship between different script stages of the same Chinese characters is built on the set of components. All components are represented by using the 2D contour formation, and the correspondence relationship between components has been constructed in the special knowledge database. In the end, the evolution animation of Chinese characters is generated by using the 2D contour morphing technology.

The main contribution of the paper firstly is that a complete Chinese character teaching system is constructed, including the knowledge databases, the key technologies and the user experience study. Secondly, the system automatically generates the evolution animation on the screen, when given two or several writing stages of the same characters. In contrast with the flash animation, the system avoids building the evolution animation for each Chinese character in the different writing stages, as a result of using the Chinese character structure theory. Finally, some key technologies in the system is proposed, including the strokes extraction, the component modeling, the Chinese character parsing and the Chinese character morphing.

We choose two representative scripts in the experiment, i.e. seal script and clerical script. The seal script is called ancient script, while the clerical script is known as modern script. The frequently used Chinese characters in seal script and clerical script are used as test data of the system and several evolution animations of the Chinese characters are generated by using the system.

The rest of the paper is organized as follows. Firstly, the related work is discussed, and then the Chinese character teaching system is introduces briefly. Moreover, the strokes extraction, the Chinese character parsing and the Chinese characters morphing are described in detail. Finally, the experimental results and the conclusion are presented.

## Related Work

Almost all researches of Chinese character focus on the character recognition, including the offline handwriting recognition, online handwriting recognition and the optical character recognition (OCR). Some key technologies are discussed in the following.

The stroke extraction is very important step in the research of Chinese character. Most existing methods for stroke extraction use thinning process. Before performing the stroke extraction task, all strokes in a Chinese character have been cut into several line segments from crossing and touching points. Then all line segments are tested by some decision rules to reconstruct the original stroke correctly in the stroke extraction module. For example, [4], [5] use thinning algorithm to obtain character skeletons as the start of stroke extraction. Some other approaches extract the stroke by using other kind of stroke information, such as contour or line adjacency graph (LAG). The approach proposed in [6] uses the feature points of the outer contour of the Chinese characters. They compute the K- curvature of the contour points that defined in their paper to find the convex points and concave points of the contour. Because the position where strokes intersect, the position where the end of one stroke attaches to that of another stroke, and the position of the inner side of turning stroke corner will have concave pints or convex points. Then some laws are used to find the relevant concave points by tracking the contour points, break the contour in the concave points, and combine two or more contour fragments by connecting the concave points which fits the laws to be a stroke. However, there are many ambiguous zones in the process of stroke extraction, due to the crossing between strokes in Chinese characters.

For the character recognition, there are two methods to represent and analyze the Chinese characters, i.e., the statistical method and the structural method. The statistical methods usually process the Chinese characters as a feature vector so that their similarity can be calculated by the distance between two vectors. Various statistical methods have been proposed for Chinese characters recognition, such as k-nearest-neighborhood classifier [7], K-Means clustering and Gaussian distribution selector [8], nonlinear active shape models [9], contextual vector quantization [10] and Mahanalobis distance [11]. The structural methods extract the structure information from the Chinese characters which emphasizes the relationship between strokes. Chinese characters are often decomposed into strokes to analyze. The stroke and theirs relation information both are used to distinguish two Chinese characters. A combined method is also proposed for the character recognition. In this method, the stroke is represented by using the distributions of its length, position and angle [12]. The position and the shape of strokes are statistically modeled and their distributions are estimated from training samples. This statistical modeling is adopted to represent both statistical and structural information of characters. The MRF-based method proposed by Zeng [13] makes use of the energy of cliques to measure the similarity between the target character and the model character. It provides a much more enriched vocabulary to describe character structures.

To get outstanding animation of Chinese characters, this paper also focuses on 2D morphing technology. In general, morphing problem includes two steps. The first step is to establish the correspondence between two models. The second step is the interpolation of corresponding models. The correspondence is extremely important in these two steps. As 2D contour can well retain the appearance of Chinese characters, so the morphing problem in this paper is based on 2D contour. For the correspondence problem, a collection of 2D correspondence methods were developed in [14]–[18]. These methods can be divided into two categories. One is to establish correspondence based merely on geometry properties of the shapes [14], [15]. The other one adds some user interactions [16]–[18] to establish the semantic correspondence for satisfy human perception, then the further treatment is still based on the geometry properties. All these methods compute the correspondence by using the optimization techniques, such as, dynamic programming [14], [16], graph cuts [17], etc.

## Chinese Character Teaching System

### Ethics Statement

The study was approved by the Human Research Ethics Committees for Non-Clinical Faculties, in the Beihang University and the Beijing Normal University. The proposed system was supported by State Key Laboratory of Virtual Reality Technology and System, Beihang University. The data of different writing stages of Chinese characters were obtained from the School of Chinese Language and Literature, Beijing Normal University. The all of authors from the two institutions declare that there is no actual or potential conflict of interest in this paper. In the study of user experience, there were 102 students and 5 Chinese character teachers who volunteered to participate in the experiments. This experiment was affirmed to cause no potential injury.

### Chinese Character Structure Theory

The Chinese character structure theory provides the systematically theoretical foundation for describing Chinese characters [1], which is proposed by Ning Wang based on the “六书” (Liushu). The theory uses the components to analyze Chinese character. The elements of Chinese writing system are not characters but components which bring certain meanings, when they were used to compose Chinese characters. The vast majority of Chinese characters are phono-semantic compounds, with a semantic component giving a broad category of meaning and a phonetic component suggesting the sound. For example, those Chinese characters “桃” (peach), “梨” (pear), “桔” (orange), “桂” (laurel), “梅” (plum), which have a common semantic component “木” (wood), are all used to record a name of woody plants, when the phonetic components are different.

**Figure 2:**
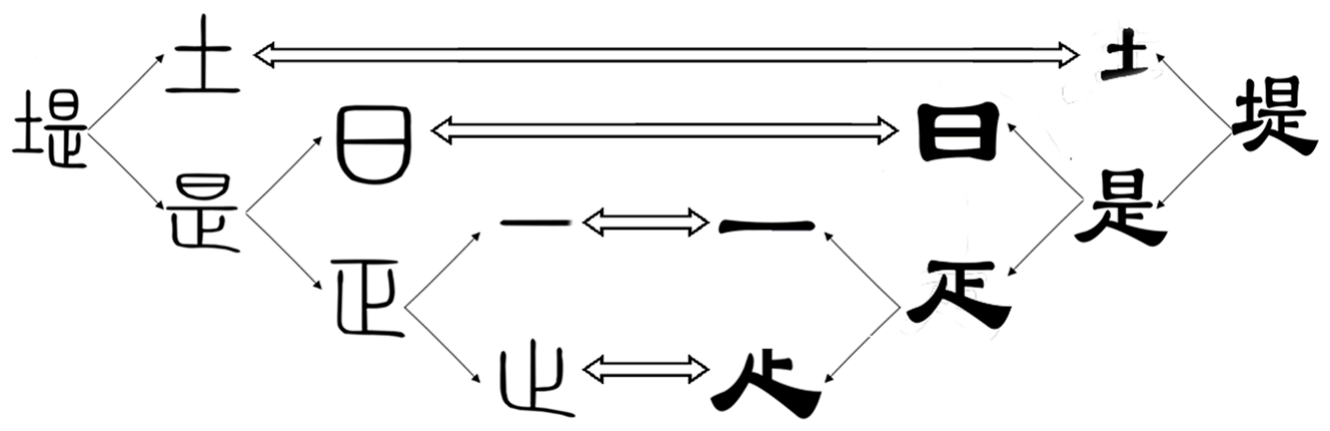
The hierarchical components of Chinese Character “堤” (ti) and their correspondence in different script. The seal script of ti and its components are shown in the left, and the clerical script of ti and its components are shown in the right. The arrows indicate the correspondence between these components.

There are two types of components. One type is the primitive component, it is not composed by other components, such as “
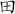
”, “力”, etc. The other type is compound components, it is composed by other components, such as “
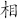
” (“木” +“
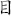
”), “
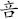
” (“立” + “
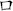
”), etc. Therefore, a Chinese character is composed of components hierarchically. If a Chinese character is composed by only one component, i.e. the Chinese character itself, then the Chinese character is called non-composite character. Usually, a Chinese character composed by no less than 2 components is called composite Chinese character. For example, the Chinese character “
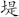
” is composed of the “土” and the “
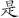
”, as shown in Figure 2.

In the evolution process of Chinese character, the shape and topology have had the huge variation at different writing stages. For example, this legibility stems from the highly rectilinear structure, the structure tends to be square, and so on. But the forms and meanings of Chinese character is preserved and passed down by using the components and the component functions. So, all the Chinese characters on the same historical diachronical level have their own component elements, and all the Chinese characters on the different historical diachronical level are connected into a sign system by using the semantic relevance between components. For example, Figure 2 shows the correspondence between components of Chinese character “堤” in differen scripts.

### System Architecture

**Figure 3:**
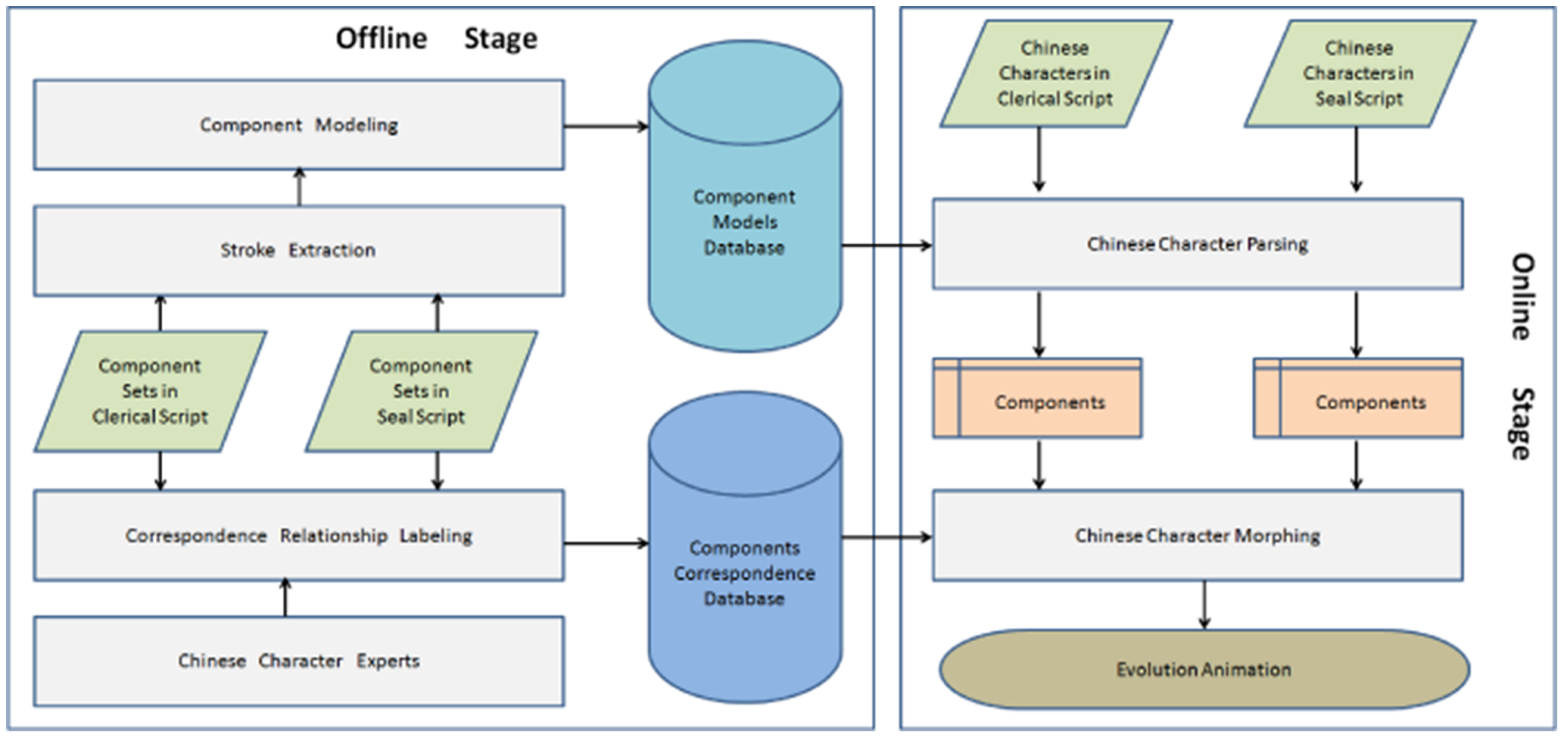
An overview of the Chinese character teaching system.

Based on the Chinese character structure theory, the Chinese character teaching system is proposed. The teaching system includes two main phases, i.e. the offline phase and the online phase. Figure 3 shows the system architecture and the relationships between technologies.

The offline phase implements the digital representation of the Chinese character structure theory and constructs the knowledge database, including the component models database and the components correspondence database. The component models database mainly is used to store the digital representation of the component for each script. Based on the component models database, we can parse the input Chinese character into a set of component, as shown in Figure 2. To this end, we input the binary images of components, extract the strokes in given components, and represent the strokes and their relationships by using the statistical structure method. All above information are stored in the component models database.

The components correspondence database is used to store the semantic relevance between components from the different scripts. Then these correspondence will be used in the process of Chinese character morphing. To this end, the experts of Chinese character are invited to build the sematic correspondence between components from different script, and they use the interactive labeling tool to manually label the rough correspondence based on the extracted strokes. Finally, the fine correspondence will be established automatically by using the interpolation on the key points.

In online phase, the input Chinese characters firstly are split into components by matching the extracted strokes with the component models from the component models database. Then, the correspondence between components is searched and built by using the components correspondence database. Finally, the evolution animation of Chinese characters is generated by morphing method. Therefore, the online phase mainly includes two key modules, i.e., the Chinese character parsing and the Chinese character morphing. The Chinese character parsing is used to split the given Chinese character into components, while the Chinese character morphing is to generate the evolution animation. To implement the system, four key technologies are proposed in the system, i.e., the stroke extraction, the component modeling, the Chinese character parsing and the Chinese character morphing. We will introduce these key technologies in the following sections, respectively.

## Stroke Extraction

### Singular Region Detection

Due to the cross between strokes in Chinese characters, there are many ambiguous zones called as singular region in the stroke extraction. We propose a novel method to detect the singular region.

**Figure 4:**
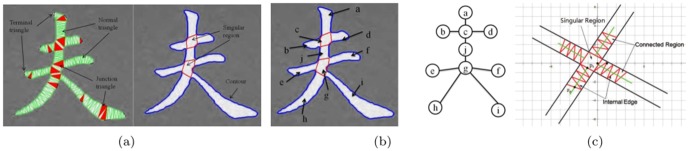
The key steps for stroke extraction in a Chinese character. (a) The shape representation of Chinese character fu by using the triangular mesh and its singular regions. (b) The Graph representation of Chinese character fu and its sub-strokes (a,b,d,j,e,f,h, and i) and singular regions (c and g). (c) The connected region of the sub-stroke and the estimation of its tangent direction.

Given a Chinese character, its contour and corners are extracted. Then the contour is evenly divided into a set of discrete points by uniform sampling between any two corner points. Based on the discrete points, the Chinese character is represented as a set of triangular meshes by using the Constrained Delaunay Triangulation (CDT) [19]. An example is shown in Figure 4 (a, left). According to the number of internal edges, there are three types of triangle meshes in the representation. The junction triangle owns three internal edges, while the normal triangle owns two internal edges and the terminal triangle owns one internal edge. We merge the junction triangles to locate the singular region in the Chinese character.

We use the point to boundary orientation distance (PBOD) curve [20] to merge the junction triangles. The PBOD curve is a vector, and the 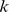-th value in the vector is the distance from the central point of triangle to the boundary along the 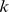 -th quantized orientation, where 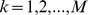 and 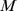 is an integer that denotes the quantization number from 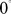 to 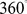. The number of the crests in the PBOD curve is used to determine the degree of the central point of junction triangle. If 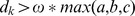, a crest is determined, where 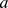, 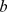 and 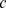 are edges of junction triangle, 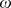 is a constant value. If degree of the central point of junction triangle is less than 3, the junction triangle is a spurious junction triangle. Then we delete it from the junction triangle set. We compute the distance between central points of two junction triangles, 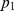 and 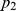 by using the equation 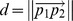. If 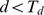, the two triangles need to be merged. When all junction triangles are merged, the singular region is generated, as show in Figure 4 (a, right).

### Stroke Search

After all the singular regions are detected, a Chinese character is divided into two types of regions, i.e., the sub-strokes and the singular regions. The sub-strokes are defined as a segment separated by a series of singular regions and a stroke is considered as the concatenation of sub-strokes and singular regions.

We build an undirected graph 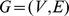 to model each Chinese character [21], where 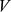 and 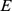 are the sets of the nodes and edges. Each node in 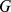 represents one of the sub-strokes or singular regions of the character, as shown in Figure 4 (b). A node represents a singular region, while its degree is equal to or more than three. Similar, a node represents a sub-stroke, while its degree is less than three. According to the degree information, sub-strokes can be divided into three types: jointed with singular region at one end (degree 1), at both ends (degree 2), and without any jointed singular region (degree 0).

We utilize the continuity analysis of sub-strokes to determine whether a pair of sub-strokes jointed with the same singular region will be contiguous into a stroke. The local continuity analysis is defined as follows:

#### Definition 1

*Given a pair of sub-strokes* 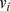 *and* 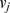, *jointed with the same singular region* 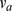. *If* 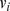 *and* 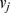 *belong to the same stroke and their features are continuous, then the pair of* 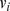 *and* 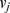 *is continuous at* 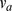, *denoted as* 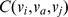 *or* 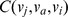.

#### Definition 2

*Given a sub-stroke* 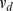 *jointed with a singular region* 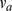. *If there is no sub-stroke* 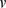 *satisfying* 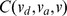, *then* 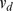 *is terminated at* 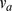, *denoted as* 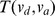.

#### Definition 3

*Given a sub-stroke* 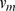 *jointed with a singular region* 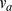. *If there are* 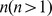 *sub-strokes* 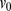, 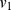, 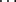, 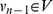, *satisfying* 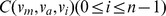, *then* 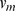 *is multi-traced at* 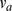.

There are three features of the connected region are used in the process of continuity analysis. The connected region is a part of the sub-stroke and close to the singular region, as shown in Figure 4 (c). The mean width 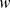 of the connected region is approximated as:
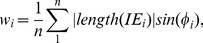
(1)where 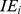 is the internal edge that in the connected region, 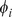 is the angle between 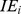 and contour, 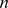 is the number of the 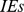.

The orientation angle of the connected region is defined as:
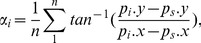
(2)where 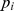 is the mid-point of 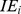 and 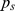 is the start point of the connected region.

The curvature of the connected region is represented by the orientation angle of the contour, is defined as:
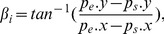
(3)where 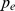 are end point of the connected region.

After computing the three features above, we estimate the continuity of two sub-stroke 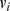 and 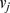 which jointed with the same singular region 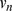 using the function below:
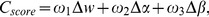
(4)where 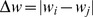 is the width difference, 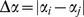 is the angle difference in medial axes, 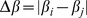 is the gradient difference in the contours of sub-strokes. 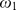, 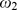 and 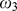 are the weight for 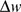, 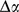 and 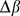 respectively.

Once the continuity of sub-stroke has been computed, the problem of stroke extraction is transformed to that of searching all the simple paths in the Graph 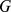 with a certain number of conditions satisfied.

A simple path 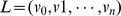 in 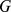 is 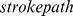, if the following two conditions are satisfied:

**Condition 1**: if 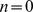, 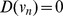, i.e., 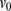 is an isolated node (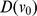 represents the degree of the node 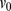);

**Condition 2**: if 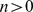, two constraints below should be used. (a) **End constraint**, (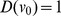 or (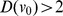 and 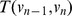)) and (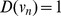 or (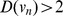 and 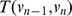)); (b) **Nod-end constraint**, For each 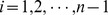, 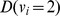 or (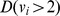 and 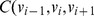)).

So, there are four stroke paths in Figure 4(b), i.e., (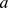, 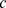, 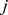, 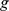, 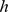), (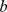, 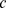, 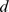), (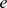, 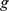, 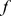), and (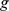, 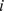).

## Component Modeling and Chinese Characters Parsing

To present the evolution of Chinese Characters in different scripts, the Chinese character is parsed into the components by using the stroke-based representation. The component models database need be built in the offline phase. Then a Chinese character is decomposed into the components in the online phase. We introduce the component modeling and the component recognition, respectively.

### Component Modeling

We construct the component model by using the statistical structure modeling method. The strokes of the component are extracted by using the strokes extraction algorithm introduced in Section 4. The component model is assumed to obey the multivariate normal joint distribution 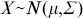. Given a component and its stroke set, 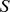, the component model 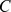 is defined by using the joint probability of all strokes as follows: 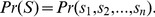
(5)where 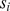 represents the stroke.

The joint distribution contains all information about the strokes and their relationships. Due to the time complexity and the proper accuracy, we tend to select the necessary relationship to simplify the model by considering the importance of neighbor relationships. The joint probability is transformed to conditional probability multiplied together as follows:
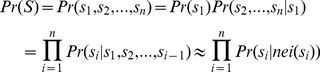
(6)where 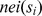 represents the neighbors of 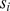.

We select the most important relationship by minimizing the Kullback-Leibler measure. The more details of derivation process can be found in reference [12]. At last, the component model are stored by using the geometrical and neighbor information of every stroke in the component.

### Chinese character Parsing

The whole stage of parsing in a Chinese character is divided into two sub-stages, i.e., single component matching and all components matching. The first sub-stage can be solved by heuristic search algorithm to find out the best set of components in the input character. The second sub-stage is categorized as the variant knapsack that demands us to find the best combination of the components found by the first stage.

#### Single Component Matching

**Figure 5:**
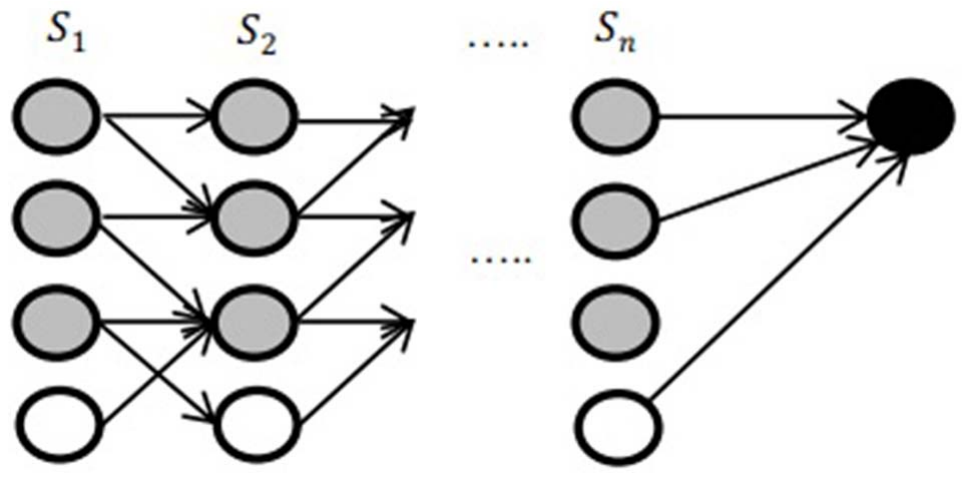
Finding the optimal matching between the extracted strokes and the component models.

In this stage, a matching result is defined by a set of correspondences between the strokes and the component models. The matching stage is illustrated in Figure 5. The 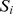 represents the strokes in the component model. The gray nodes represent the stroke in the Chinese character. The white ones show that there is not the available matching. The last black node records the final result of this matching process. The component will be selected if the matching result is greater than current best record, otherwise, discarded.

During the stroke matching process, the conditional probability is considered. For example, there are two input strokes 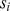 and 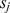, whose matching goal are 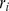 and 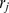, respectively. The matching probability are calculated by using the conditional probability, if they satisfy the following conditions: (a) 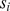 is already matched with 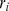; (b) 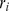 is the neighbor to 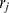. Otherwise, the probability in next matching stage is calculated without the conditional probability.

#### All Components Matching

We modify the matching result to find the most possible components. Especially, the situation of using the reduplicate components in a Chinese character needs to be processed, such as, “**磊**”, “**炎**”. In a Chinese character, not only the possible components are different in the composition of strokes, but also the unselected strokes in the input characters are minimized. We regard this optimal problem as variant knapsack problem. The knapsack's size is equal to the size of strokes in input character. Every matching result has the attribute to describe the usage of input strokes. Through the solution of variant knapsack problem, we find the best combination in matching result produced by the single matching process to fulfill the knapsack. The goal is to make the collision of stroke usage as less as possible.

## Chinese Characters Morphing

### Compatible Triangulations and Optimization

After the components are extracted, the correspondences between the components are searched from the components correspondence database. With the components correspondence, the morphing sequences are generated. As the simple linear interpolation method generally yields distortion and shrink in the intermediate shapes, we use the intrinsic method proposed in [22], in which interpolated entities are compatible triangulation rather than the Cartesian coordinates of its vertices.

**Figure 6:**
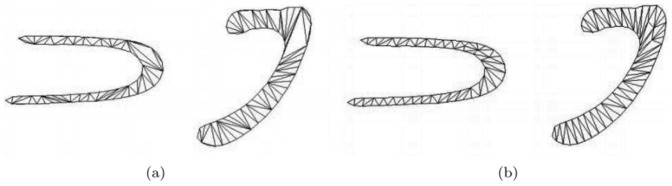
The results of compatible triangulations and compatible optimization.

For obtaining the compatible triangulation, the fine strokes correspondence firstly are computed based on components correspondence. A stroke is a closed contour and it has a start point and an end point. When the start point and the end point of two strokes are corresponded rightly, the rest of the contour points also are corresponded by using the sampling principle. The points are used to construct the triangulations by applied the minimum link interior path method [23], are shown in Figure 6 (a).

The quality of compatible triangulations is unlikely to be acceptable. Therefore, optimization techniques must be applied to improve the quality. We simultaneously use the edge-flips, the mesh smoothing and the mesh refinement to generate the high quality compatible triangulations with a small number of triangles [24]. The optimization result is shown in Figure 6 (b).

### Path Interpolation

After optimization of compatible triangulations, the problem is transformed into the interpolation problem. In single triangle case, let the source vertices be 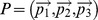 and the target vertices be 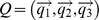, where vertices with the same index correspond. An affine mapping represented by matrix A transforms P into Q: 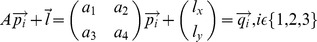
(7)


Assuming the intermediate state 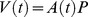, we can define: 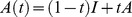. The basic idea to solve this function is factoring 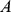 into rotations and scale-shear parts. The decomposition can be deduced as follows: 
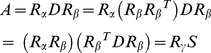
(8)


Where 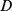, 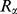 and 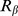 are reduced from the SVD [25], 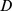 is a diagonal matrix, 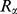 and 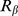 are two unitary matrices. We can compute 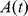 by linearly interpolating the free parameters in above factorizations: 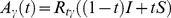
(9)


We now consider a triangulation 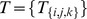 rather than a single triangle. Each of the source triangles 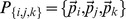 corresponds to a target triangle 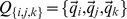. For each pair of triangles, we compute a mapping 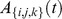. Since most of the vertices belong to more than one triangle, a mapping of all vertices could not satisfy all the individual ideal transformations 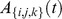. We define an intermediate shape 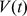 as the vertex configuration which minimizes the quadratic error between the actual matrices 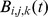 and the desired ones 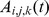. This quadratic error functional is expressed as 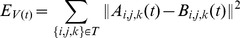
(10)where is the 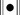 norm. In order to have a unique minimizer to 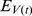, we predetermine the location of one vertex, say 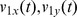. So the functional 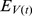 can be expressed in matrix form.
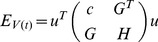
(11)where 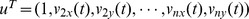, 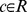 represents the constant, 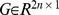 the linear, and 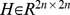 the mixed and pure quadratic coefficients of the quadratic form 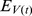. The minimization problem is solved by setting the gradient 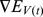 to zero. And we can invert 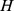 and find solutions for time 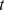 computing the corresponding 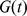 and a single matrix multiplication:



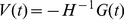
(12)


## Experiment and Results

In this section several evolution animations of Chinese characters are generated by using the proposed system. We choose two representative scripts in the experiment, i.e. the seal script and the clerical script. The seal script is an ancient style of Chinese script. It evolved organically out of the bronze script, arising in the Warring State of Qin. The Qin variant of seal script became the standard and was adopted as the formal script for all of China in the Qin dynasty, and was still widely used for decorative engraving and seals in the Han dynasty. Ever since, its predominant use has been in seals, hence the English name. Most modern-day Chinese people cannot read seal script, so it is generally not used outside the fields of seals and calligraphy. But, the emergence of seal script laid the foundation of modern Chinese characters structure and became the watershed between ancient scripts and modern scripts.

The clerical script also is an archaic style of Chinese scripts which evolved in the Warring States period to the Qin dynasty, was dominant in the Han dynasty, and remained in use through the Wei-jin periods. Due to its high legibility to modern readers, it is still used for artistic flavor in a variety of functional applications such as headlines, signboards, and advertisements. This legibility stems from the highly rectilinear structure, a feature shared with modern regular script 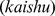. In structure and rectilinearity, it is generally similar to the modern script; however, in contrast with the tall to square modern script, it tends to be square to wide, and often has a pronounced, wavelike flaring of isolated major strokes, especially a dominant rightward or downward diagonal stroke. Some structures are also archaic.

The frequently used Chinese characters are contain in the system wrote with above two scripts. 543 components in seal script and 527 components in clerical script are digitized for constructing the component model database. In the component correspondence database, the sematic relevance between components from two scripts is assigned by the experts of Chinese characters. For the rough correspondences on the components, average 10–15 key points are manually labeled on the components by use the interactive labeling tool.

Based on these databases, the experiment consists of two parts. 1) In the system test, we quantitatively evaluate the key module in the proposed system, and correctness of component modeling and character parsing; 2) In the user experience study, we invite two types of groups to use and evaluate our system.

### System Test

**Figure 7:**
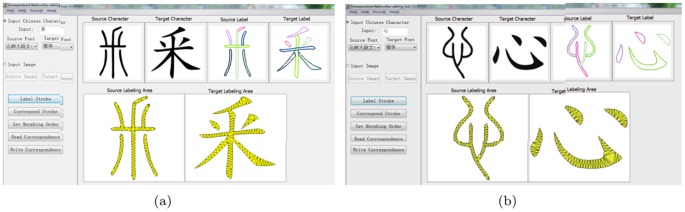
The interface of component correspondence labeling.

**Figure 8:**
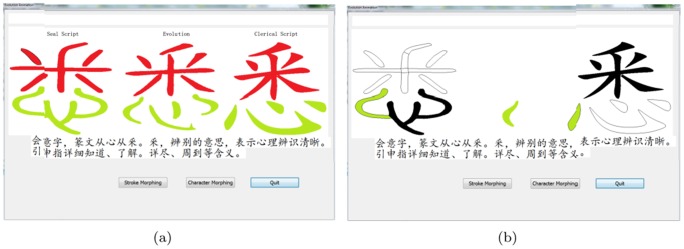
The interface of evolution animation.

The system is implemented on a PC with Intel Core 2.66 GHz processor and 4.00 GB memory by using the development environment of Visual C++6.0. The main module interfaces are showed in Figure 7 and Figure 8.

**Figure 9:**
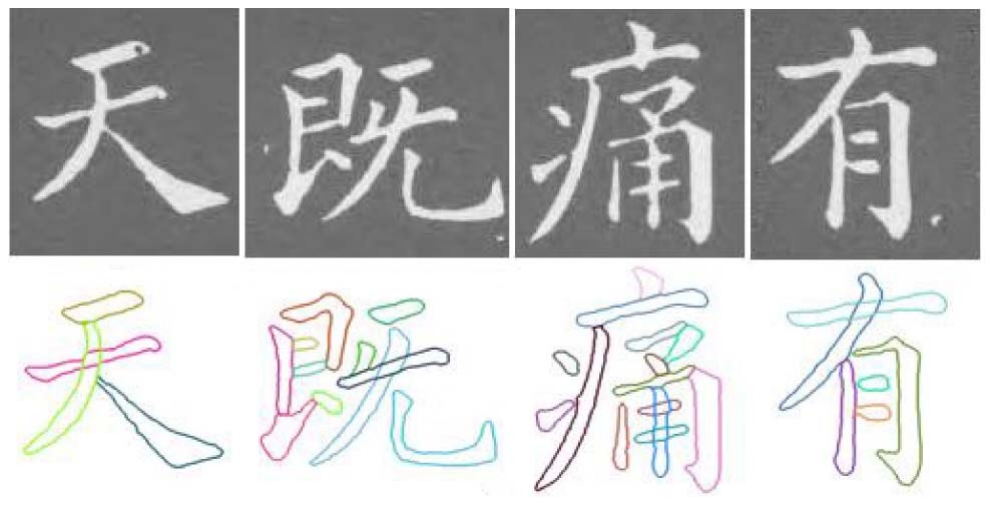
Some examples of stroke extraction for the Chinese characters.

The module of stroke extraction first is evaluated. We collect and select 1000 Chinese characters as the test objects. These Chinese characters are written by different artists with the different scripts. By the experiments, we find that the singular regions detection 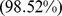, the continuity analysis 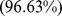 and the stroke extraction 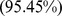 all obtain the prominent results. These higher accuracy will ensure the good results in the subsequent steps. Some examples of stroke extraction using our proposed method are shown in Figure 9.

The experiment of component recognition and parsing also is tested in the Chinese character. We compare our method with representative works on component recognition. The experiments are executed on two datasets, i.e., the test 1 includes 500 Chinese characters in different scripts, while the test 2 has 1000 Chinese characters. The compared method is the nonlinear PCA method and the stroke-based method [26]. The comparison results are shown in Table 1.

**Table 1:** The results of component recognition.

Method	Test 1 (%)	Test 2 (%)
Our Method	96.8	92.6
Nonlinear PCA	96.4	89.3
Stroke-Based	92.6	86.4

**Figure 10:**
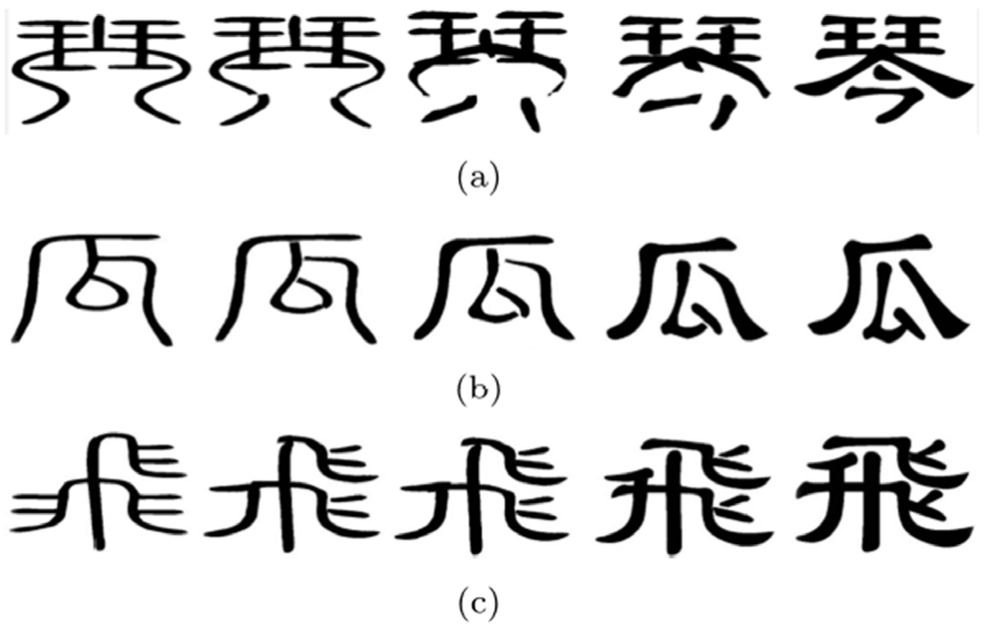
The different types of evolution animation of Chinese characters.

Finally, the evolution animation is generated fast base on the component correspondence. The fine correspondence process of each example takes less than 2 seconds in all the experiments. The intermediate shapes can be computed in real time. We can generate more than 25 intermediate shapes per second for all examples shown in this paper. As the topology of the Chinese characters are rather complicated, there are mainly three kinds of evolution sequences among Chinese character: (a) The number of components and strokes become more in the process of evolution; (b) The number of components and strokes between Chinese characters are the same; (c) The number of components and strokes become less in the process of evolution. Some examples are shown in Figure 10.

To evaluate the performance of our system, we invite two types of persons to use and evaluate our system from different perspective. The one type is the Chinese character teachers who have rich experience about the ancient Chinese character. The database of components correspondences is very important in the system, it mainly depended on the knowledge of Chinese character teachers. So, these teachers are ask to use the correspondence labeling tool to construct the database. After that, they were asked to complete a questionnaire with designed questions to evaluate the labeling process and the morphing result. The questions are listed in Table 2.

**Table 2:** The questionnaire of user experience study on the experts group.

Questions	Choices				
The system is useful for the teaching	Very	Somewhat	Neutral	Rarely	Not at all
The system will be recommended to other teachers	Very	Somewhat	Neutral	Rarely	Not at all
The system will be used in your class	Very	Somewhat	Neutral	Rarely	Not at all
The labeling is easy for the correspondence	Very	Somewhat	Neutral	Rarely	Not at all
The evolution is consistent with teaching idea	Very	Somewhat	Neutral	Rarely	Not at all

**Figure 11:**
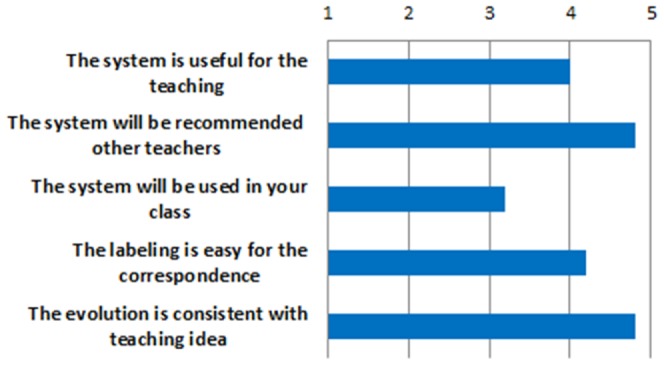
The result of expert questionnaire.

For each question, there are five choices, the corresponding scores are 5,4,3,2,1 which respectively means Very, Somewhat, Neutral, Rarely and Not at all. The average score of each question is show in the Figure 11. We can see that the experts gave higher scores in the first two questions and the last two questions but trend to be neutral in the third question. That result shows that the proposed system is effective in the process of ancient Chinese character teaching.

### User Experience Study

The another type is the students from the Beijing Normal University and the Beihang University. 102 students is invited in the age group of 20–30, 76 female and 26 male. Especially, there are 10 foreign students in our experience. The majority of students are familiar with the common Chinese character in the regular script, but they almost do not know the corresponding ancient script, not to mention the evolutions between different scripts.

**Figure 12:**
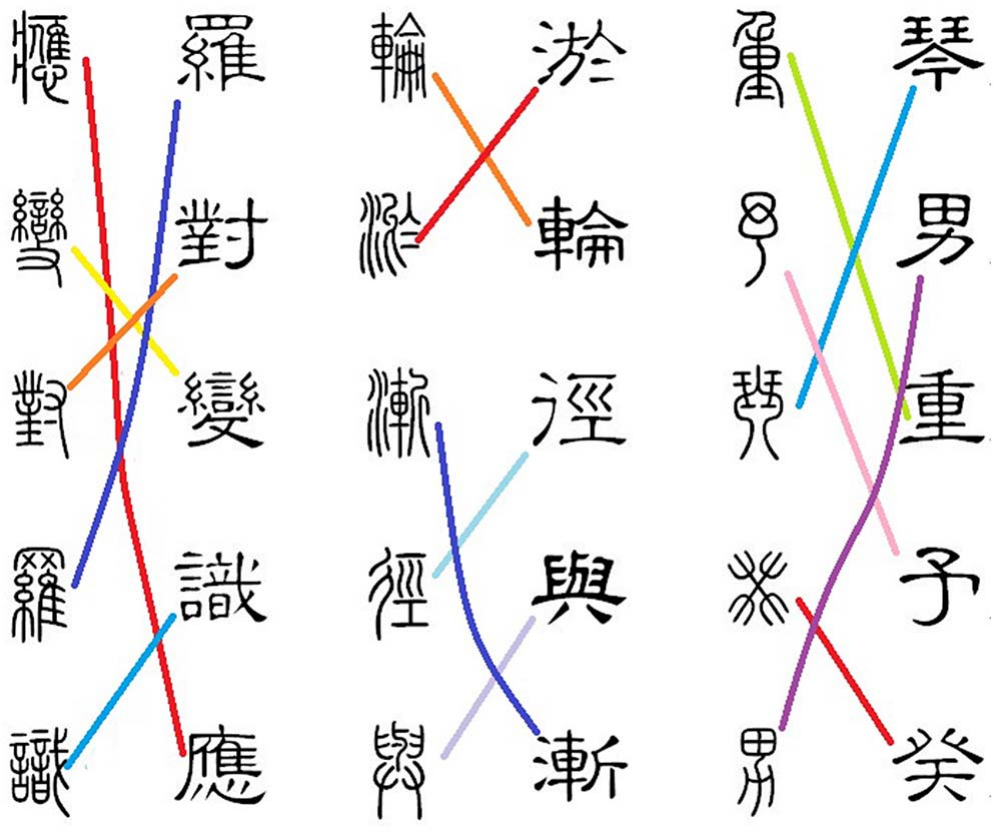
The LinkGame of Chinese characters.

To evaluate the performance of teaching system, each student learn the Chinese character by using our system. These students first watch the morphing of selected character from the system, then they are asked to do a test which is called LinkGame of Chinese characters, as showed in Figure 12. In LinkGame, a set of common Chinese characters are selected and averagely each group has about 7–10 Chinese characters. Different groups can have the repeated Chinese characters. The Chinese characters of each group have two writing stages, i.e., the seal script and the clerical script. After learning the set of Chinese characters, we require that these students link the same Chinese characters in different scripts by using the line in each group. Moreover, these students also are randomly selected into an experiment group and a control group, and each group contained the same students. The students in the experiment group are trained with the proposed teaching system, while those in the control group were trained by self learning. Compared with the control group, the average accuracy and the average speed in the experiment group are improved.

**Figure 13:**
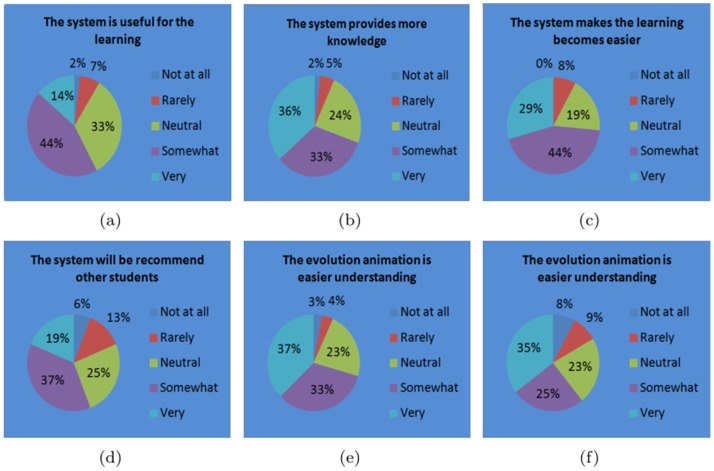
The result of student questionnaire.

After the completion of learning, the students were asked to complete a questionnaire with designed questions. There are five choices for each question to evaluate the effect of the proposed system. The questionnaires are listed in Table 3. The students responses to the questionnaire are shown in Figure 13. It can be observed that 58% students think that the proposed Chinese character teaching system is useful for their learning, while only 9% of the students showed negative opinions. Moreover, 69% of the students think it is very or somewhat interesting and 55% of the students are willing to recommend other students to try this system. In addition, 74% of students think that it is easier to study ancient Chinese by this system. In a word, the proposed system on ancient Chinese character teaching is deemed useful and interesting for most students.

**Table 3:** The questionnaire of user experience study on the students group.

Questions	Choices				
The system is useful for the learning	Very	Somewhat	Neutral	Rarely	Not at all
The system provides more knowledge	Very	Somewhat	Neutral	Rarely	Not at all
The system makes the learning become easier	Very	Somewhat	Neutral	Rarely	Not at all
The system will be recommended to other students	Very	Somewhat	Neutral	Rarely	Not at all
The evolution animation is easier understanding	Very	Somewhat	Neutral	Rarely	Not at all
The evolution animation is more smooth	Very	Somewhat	Neutral	Rarely	Not at all

## Conclusion and Future Work

In this paper, we propose a novel Chinese characters teaching system by generating the evolution animation to show the evaluation between different scripts of the same Chinese characters. The teaching system is based on two important knowledge databases, i.e., the component models database of each script and the components correspondence database for different scripts. Moreover, four important technologies of the system are implemented for generate the evolution animation automatically, including the stroke extraction, the component modeling, the Chinese character parsing and the Chinese character morphing. Finally, the teaching system is tested on two representative scripts and the user experience studies are reported.

However, there are many places need to improve in the teaching system, including to reduce the user interaction and to introduce the machine learning methods. Meanwhile, the interpolation methods should be improved for the distortion and shrink still appears in some cases. In future, the feedback study of the user experience study will be strengthened, the components knowledge database will be enriched, and the teaching system will be promoted in the teaching process.
